# Reduced Dietary Protein Induces Changes in the Dental Proteome

**DOI:** 10.1002/jezb.70004

**Published:** 2026-01-08

**Authors:** Robert W. Burroughs, Christopher J. Percival, Natasha S. Vitek

**Affiliations:** ^1^ Department of Ecology and Evolution Stony Brook University Stony Brook New York USA; ^2^ Center for Inclusive Education Stony Brook University Stony Brook New York USA; ^3^ Department of Anthropology Stony Brook University Stony Brook New York USA; ^4^ Department of Biology Bradley University Peoria Illinois USA

## Abstract

Experimental studies have demonstrated that nutritional changes during development can result in phenotypic changes to mammalian cheek teeth. This developmental plasticity of tooth morphology is an example of phenotypic plasticity. Because tooth development occurs through complex interactions between manifold processes, there are many potential mechanisms which can contribute to a tooth's norm of reaction. Determining the identity of those mechanisms and the relative importance of each of them is one of the main challenges to understanding phenotypic plasticity. Quantitative proteomics combined with experimental studies allow for the identification of potential molecular contributors to a plastic response through quantification of expressed gene products. Here, we present the results of a quantitative proteomics analysis of mature upper first molars in *Mus musculus* from a controlled feeding experiment. Pregnant and nursing mothers were fed either a low‐dietary protein (10%) treatment diet or control (20%) diet. Low‐dietary protein was not associated with reduced molar size or skull length. However, expression of tooth‐related proteins, immune system proteins, and actin‐based myosin proteins were significantly altered in our low‐dietary protein proteomics sample. The differential expression of immune proteins along with systematic reduction in actin‐based myosin protein expression are novel discoveries for tooth proteomics studies. We propose that studies that aim to elucidate specific mechanisms of molar phenotypic plasticity should prioritize investigations into the relationships between IGF regulation and tooth development and actin‐based myosin expression and tooth development.

## Graphical abstract

Low dietary protein (10%) from normal (20%) does change protein expression in tooth proteome and alter developmental pathways. Among the significant protein expressions changes are actin‐based myosins, tooth, and bone development proteins. Perplexingly tooth size is not altered, suggesting more nuanced phenotypic response to low dietary protein in early development.

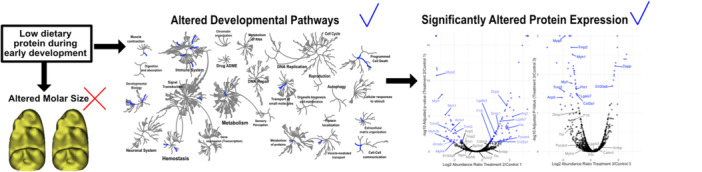

## 1 Introduction

Phenotypic plasticity, the differential expression of a phenotype, is often invoked as a way that organisms respond to changing environments. Within lab and common garden experiments, nutritional changes such as changes in the quantity of dietary protein induce plastic changes to mammalian cheek tooth phenotypes (Patton and Brylski 1987; Paynter and Grainer 1956; Shaw and Griffiths 1963). Typically, these are changes in tooth size, size and shape of tooth cusps, and timing of eruption (Holloway et al. 1961; Patton and Brylski 1987; Paynter and Grainer 1956; Searle 1954; Shaw and Griffiths 1963). This demonstrates that there is plasticity in dental development that can provide a short‐term, nonevolutionary response to changing environments (Levis and Pfennig 2021). However, measures of plastic phenotypic response to environmental change often do not identify the molecular and genetic pathways underlying those changes. The use of quantitative proteomics to quantify differences in protein expression could allow for the identification and study of pathways that are altered by an environmental change (e.g., are plastic). Here, we present a study characterizing protein expression variation in a sample of upper first molars (M1s) and phenotypic variation in lower first molars (lower M1s) from lab mice fed a low‐protein diet as part of a controlled feeding experiment. This experiment was designed to induce developmental changes to dental traits and to identify proteins and associated pathways that may underlie phenotypic plasticity. This allows us to characterize those pathways most impacted by exposure to low dietary protein during embryonic and early postnatal tooth development.

## 2 Proteomics

Proteomics is broadly the field focused on identifying, annotating, and quantifying variation of proteins. Quantification of variation includes both protein sequence variation and protein expression variation. Informatics approaches are applied to protein spectral data collected via tandem liquid‐chromatography mass‐spectrometry (LC‐MS/MS) (Heck and Neely 2020). Protein expression profiles are typically tissue‐ and developmental stage‐dependent and must be interpreted within the specific spatiotemporal contexts in which samples were collected (Rebeaud et al. 2021).

An assemblage of proteins that is expressed within a specific tissue or structure is often referred to as a “proteome” (Sharma et al. 2020). For example, within the dental proteome enamel and dentin forming proteins are found in high abundance (Sharma et al. 2020). However, outside of dental tissues, these proteins are detected in low abundance and only at certain developmental stages (Bansal et al. 2012; Ritchie 2018).

If basic processes of tooth mineralization are impacted by dietary deficiency, we expect a change in associated protein expression and a change in phenotype such as occlusal pattern, size, and/or shape (Harjunmaa et al. 2014). It is possible that some aspects of tooth mineralization are more easily perturbed by environmental change than others and their associated pathways may more frequently underlie plastic changes. Measuring the tooth proteome may allow us to identify potential candidates underlying plastic changes in tooth phenotype.

Beyond stereotypical tooth‐associated proteins, previous characterizations of the dental proteome have suggested that teeth serve as reservoirs of more general patterns of organismal protein expression during amelogenesis (tooth mineralization) (Froment et al. 2021; Giovani et al. 2021; Green et al. 2019; Sharma et al. 2020). For example, Green et al. (2019) recovered immune system related proteins from enamel that was micro‐sampled from near the enamel‐dentin junction of mineralizing pig molars. These immune proteins currently have no known function in amelogenesis. These proteins are definitively present within enamel tissue, but they may originate in nearby salivary glands that are in contact with enamel cell proliferation zones and regions of mineralization at the enamel‐dentin junction (Green et al. 2019; Jágr et al. 2019). If mineralized teeth serve as an archive of broader organismal protein expression at the time of mineralization, it is possible that tooth proteomic data could provide evidence of more generalized responses to environmental perturbations.

## 3 This Study

To determine what pathways are altered by an impoverished diet, we conducted a controlled feeding experiment and investigated protein expression in the cheek teeth. The study presented here is part of a larger research program that will also investigate the impacts of low dietary nutrition on a variety of tooth phenotypes. Pregnant and nursing mouse dams were fed a low protein or control protein diet and effects were measured in their offspring. Previous feeding studies on dietary protein quantity in rats and mice suggested that a threshold of 10%–12% (by weight) dietary protein reduction from a control of 20%–24% should induce phenotypically plastic changes to molar size, skull length, and disrupt timing of molar development (Barbeito‐Andrés et al. 2016; Holloway et al. 1961; Miller and German 1999; Paynter and Grainer 1956; Paynter 1961; Pucciarelli 1980; Shaw and Griffiths 1963).

With this context we anticipated that reduction in dietary protein would disrupt normal tooth development and alter protein expression patterns during embryonic and early postnatal development. We predicted a significant reduction in M1 size, consistent with previous reports (e.g., (Holloway et al. 1961; Paynter and Grainer 1956; Paynter 1961; Shaw and Griffiths 1963) and significant reduction in skull length based on previous studies in rat and mouse (e.g., Barbeito‐Andrés et al. 2016; Lobe et al. 2006; Miller and German 1999; Pucciarelli 1980). Previous studies suggested that halving dietary protein would likely increase risk of infection and increase metabolic stress in low protein mice (Giovani et al. 2021; Steward et al. 2023). Thus, we anticipated that there might be additional proteomic signals related to stress or immune system function if those protein signatures are preserved within dentition. Recovery of significant differential expression of proteins because of a dietary change allowed us to identify and rank the molecular pathways most likely to be perturbed by environmental influences like dietary protein reduction.

## 4 Methods

### 4.1 Feeding Experiment

A breeding colony of inbred strain C57BL/6J (RRID: IMSR JAX:000664) mice was established at the Division of Laboratory Animal Research at Stony Brook University, in accordance with authorized IACUC protocol (SBU IACUC 2023‐0014). Male and female mice were acquired at 8 weeks of age and housed in residence to acclimate until breeding began at 12 weeks of age. Males were placed with females for up to 72 h (3 day‐night cycles) and females were checked daily for presence of a copulatory plug. Once a plug was present or 72 h had passed, the male and female were separated. Females, regardless of plug presence, were then randomly assigned to control or treatment diets to ensure that any developing embryos were on as consistent a diet as possible. Mice assigned to the control group were fed a 20% raw protein diet (PicoLab Rodent Diet 20, 5053). Mice assigned to treatment were fed a 10% raw protein diet (Mod LabDiet 5053 with ~10% Protein Red, 5BQM). Before this assignment, all specimens consumed the control diet.

If females were not pregnant, as evidenced by swollen abdomen after ~7 days postmating attempt, low‐protein females were cycled back to the control protein (20%) diet. They were re‐acclimated to that diet for 14 days before additional mating was attempted. To ensure that additional stress was not induced via single housing, pairs of females were housed together during acclimation and pregnancy. Mating timings were staggered to ensure that two females in the same cage would not give birth at the same time. This allowed for the separation of females and their litters once they had given birth.

Pregnant and nursing females in the treatment group were fed only the low‐protein diet for the remainder of their lifespan. Offspring were weaned at approximately ~21 days postnatal (P21). Siblings were housed with their respective sex and fed their respective diets until P28 to ensure full eruption of the third molars. Dams and offspring were euthanized when the offspring were P28. Dams were not reused to avoid introducing bias related to improvement of maternal care from first to second litter (Weber and Olsson 2008). A total of 12 litters (7 treatment, 5 control) constituting 74 offspring (treatment *n* = 34, control *n* = 30) from this feeding experiment were collected. Aside from the 74 collected offspring, there was postnatal attrition of one full litter of treatment pups (*n* = 8), where the mother declined to nurse the pups. The smallest pup from each of five litters (treatment *n* = 3; control *n* = 2) also did not survive to weaning.

### 4.2 Sampling

**Figure 1:**
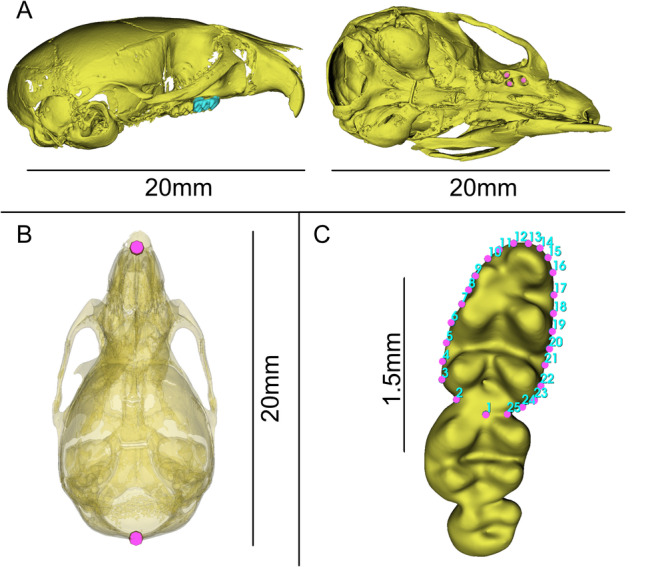
Models of skulls of treatment specimens used for proteomic sampling and processed from µCT scans. (A) Lateral and Ventral view of skull, showing where right upper M1 was removed, M2 and M3 are still visible. Light blue shows M1 still in place, Fuschia dots indicate where roots for M1 once inserted. (B) Dorsal view of skull, opacity adjusted to 50% to show location of landmarks for straight‐line skull estimate. (C) Scheme of 25 landmarks placed on left lower m1 used to assess crown area and shape between treatment and control specimens.

For proteomics data we opportunistically sampled specimens from four litters of our feeding experiment. In our sample, males were the more common sex, and thus we selected by random, four male sibling pairs for destructive sampling. Selecting only males also removed variation in protein expression that would be associated with specimen sex within our small sample. The upper first molar (upper M1) was selected for protein extraction because of its ease of extraction and because it and its lower jaw counterpart are the first molars to develop and erupt (Figure 1A). Eight upper M1s (treatment *n* = 4; control *n* = 4) were extracted from sibling pairs from four litters immediately after euthanasia by dissecting away the maxillary gingiva and exposed maxillary bone to reveal tooth roots. A blunt probe was used to lever the tooth out. Excess tissue, including any root bundle, was removed with forceps. Extracted teeth were then washed with 70% ETOH, wiped dry with a clean Kimwipe, and stored in new cryotubes. Teeth were placed into a −80°C freezer and maintained at −80°C until preparation for protein quantification.

For phenotypic data, we sampled the left lower first molar (lower M1) of specimens from four treatment and four control litters. These litters include the four litters sampled for proteomics. We measured the phenotype of 50 (treatment *n* = 24; control *n* = 26), where 24 were males and 26 were females. Three of the specimens sampled for proteomics (2 controls, 1 treatment) were excluded from the phenotypic data because they were held out for future synchrotron scanning. Specimen data are included in Supporting Information.

### 4.3 Phenotyping

We utilized µCT imaging to generate high‐fidelity 3D surfaces of mouse specimens. This approach allows us to utilize semiautomated phenotyping approaches. We batch scanned specimens at the New York Institute of Technology Visualization Center on a Bruker Skyscan 1173 at 75 kV, 106 µA, at a resolution of 19.938 µm. Specimens were decapitated post‐euthanasia and ethafoam was inserted to separate the upper and lower dentition. Heads were then wrapped in Kimwipes and stacked vertically in 50 mL Falcon tubes, with males and females grouped separately within the tube and one to two litters of the same type (e.g., all control or all treatment) per tube. Litters were separated by stacked ethafoam discs that form a visual barrier in reconstructed scan data.

Post‐processing of µCT data was done at Stony Brook University, by author RWB, utilizing 3D Slicer (version 5.9.0‐2025‐07‐04; (Fedorov et al. 2012). Reconstructed batch scans were loaded into Slicer and individual specimens were cropped from the batch scan volume. Minimum thresholding was used to segment all bony elements within the scan. The Islands segmentation tool was used to separate out the largest element within the segment, the cranium. Once segmented, crania were converted to 3D models. It was necessary to segment the entire tooth row, as opposed to isolate the lower M1, due to contact between teeth. Minimum thresholding was again applied, but with increased minima compared to bony elements to isolate the denser enamel. The Scissors tool was used to circle the lower left tooth row and “Erase Outside” was selected to remove all remaining elements within the segment. Finally, the Islands tool was again used to keep only the largest element within the segment (the tooth row). Once segmented, toothrows were converted to 3D models.

We utilized landmarks to measure phenotypic data on 3D models. To place landmarks, we utilized Automated Landmarking through Point cloud Alignment and Correspondence Analysis (ALPACA) implemented within the 3D Slicer SlicerMorph extension (Porto et al. 2021; Rolfe et al. 2021). The strength of ALPACA lies in rapid implementation and reduced user bias in landmark placement for landmark projection. ALPACA accomplishes this with a point cloud‐based deformation of a reference landmark template and mesh to match nonreference specimens. Once the deformation has occurred, landmarks from the reference specimen are projected onto the nonreference specimen. Because this method utilizes a deformation‐based approach it is necessary that specimens be similar in shape (as they are in this intraspecific study of a single tooth) and that placed landmarks be quality checked after running ALPACA. We generated two reference templates: (1) a control cranium with two landmarks for calculating straight‐line skull length, (2) a control lower M1 with 25 landmarks representing tooth outline/crown footprint (Figure 1B,C). After ALPACA was run, a single author (RWB) checked all of ALPACAs landmark position predictions and moved any landmarks that were not properly projected.

Analysis of landmark data was conducted in **R** with core R tools (RCoreTeam 2023), the **geomorph** (Adams and Otárola‐Castillo 2013) package, with visualization via the core **plot** functions and **ggplot** package (Wickham 2016). The landmark files and R script are in Supplemental Materials. To test hypotheses of changes in skull‐length we measured the line between landmarks at the apex of the foramen magnum and the junction between the maxillae (Figure 1B). A Welch's *T*‐Test was calculated using the ***t*.test** function in R to determine if there was a significant difference between treatment and control group mean skull lengths. Because our prediction was that treatment specimens would be smaller, we utilized a single‐tail alternative hypothesis, implemented in R as “greater than.”

Crown footprint area of the left lower first molar (lower M1) was represented by 25 landmarks placed around the widest portion of the crown (Figure 1C). These landmarks were used to estimate a convex hull that approximates the tooth crown area. We compared mean crown area estimates between groups using Welch's *T*‐Test, as described for skull length.

The decision to phenotype the lower M1 while quantifying the proteome of upper M1 was based on sampling constraints. Both molars develop simultaneously, rendering them as comparable as possible, while simultaneously accounting for the potential reciprocal destructive sampling of each method collection approach. Previous studies indicate that high power µCT has been shown to damage DNA and could possibly degrade proteins (Immel et al. 2016) and our proteomics protocol required destroying the entire upper M1. Choosing the lower molar for phenotyping also aids in future phenotypic comparisons because lower molars are usually found in higher abundance within paleontological samples, resulting in studies of phenotypic evolution and plasticity often emphasizing measures of lower molars (Bell and Jass 2011; Burroughs 2019; Procopio et al. 2018; Ungar 2010). We chose an outline‐based approach over traditional straight‐line measurements because crown area has been shown to be a more sensitive tool for assessing size (Hopkins 2018).

### 4.4 Protein Analysis by LC‐MS/MS

For protein analysis we first determined if our extraction and proposed sampling worked by initially sampling only two treatment and two control specimens. After validating our protocols, we sampled the remaining two treatment and two control specimens. Proteins were isolated in 5% SDS, 100 mM TEAB, 10 mM DTT using a Precellys bead homogenizer for two cycles and spun at 16,000*g* for 5 min, which pulverized the tooth tissue. Supernatants were reduced at 55°C for 30 min, and cysteines were alkylated with 25 mM iodoacetamide for 30 min at room temperature in the dark. Samples were acidified with phosphoric acid, proteins were then precipitated with 90% methanol, 50 mM TEAB, and bound to S‐Trap solid phase cartridges (Zougman et al. 2020). Protein precipitates were washed with 90% methanol, 50 mM TEAB and digested with trypsin (20 µg, Sigma‐Aldrich. Trypsin, TPCK‐treated; #4352157) at 47°C for 2 h. We ran two blank S‐Trap samples per analytical sample. No protein or peptide was recovered from blank S‐Trap samples, except for a single keratin peptide at m/z 1082. Precipitates were then sequentially eluted with 50 mM TEAB, 0.2% formic acid, and 50% acetonitrile, the 0.2% formic acid elution step was by centrifugation at 4000*g* for 1 min each. Peptides were dried by vacuum centrifugation, desalted using HLB SPE cartridges (Waters), dried and resuspended in 20 µL 0.1% formic acid/water.

Peptides were analyzed by C18 reverse phase LC‐MS/MS (Thermo Nano1200) using a 2 µL injection volume. HPLC C18 columns were prepared using a P‐2000 CO2 laser puller (Sutter Instruments) and silica tubing (100 µm ID × 15 cm) and were self‐packed with 3 u Reprosil resin. Peptides were separated using a flow rate of 300 nL/min, and a gradient elution step changing from 0.1% formic acid to 40% acetonitrile (CAN) over 90 min, followed by a 90% CAN wash and re‐equilibration steps (buffer A 0.1% formic acid/water, buffer B 90% acetonitrile/water with 0.1% formic acid). Parent peptide mass and collision‐induced fragment mass information were collected using an orbital trap (Q‐Exactive HF; Thermo) instrument followed by protein database searching using Proteome Discoverer 2.4 (Thermo). Electrospray ionization was achieved using spray voltage of ~2.3 kV. Information‐dependent MS and MS/MS acquisitions were made using a 50 ms survey scan (m/z 375–1400) at 60,000 resolution, followed by “top 20” consecutive second product ion scans at 15,000 resolution. Peptide and spectra false discovery rates were set to 0.01 and 0.05 FDR bins, with 10 ppm MS and 0.05 Da MS2 tolerances, allowing up to two missed tryptic cleavages, and a minimum peptide size of six amino acids. Variable modifications included M‐oxidation, NQ‐deamination, and ST dehydration. Peptide‐specific label free quantitation (mapping) was performed using Proteome Discoverer 2.4 (SequestHT Percolator algorithm), linking peptides to annotated mouse proteome (Uniprot mouse 16982 sequences; 4‐2019, downloaded October of 2023) and the MQ contaminant data set for standardized protein identification. Label free abundance estimations were performed (Sequest HT LFQ). Raw data files are available on the MassIVE database using identifier MSV000098600.

### 4.5 Filtering

To assess if dietary changes resulted in significantly altered protein expression, we first filtered our mapped proteomic data. Filtering was done in ProteoRE (Mehta et al. 2023). Because spectral ionization can vary between LC‐MS/MS analyses (e.g., Muntel et al. 2015; Z. Wang et al. 2024) we initially treated our data set as two distinct datasets, representing the initial sampling (Group 1) and additional sampling (Group 2) (Supporting Information). There was no expectation that Groups 1 and 2 were substantially different from one another based on the experimental procedure: groups differed only by the date of LC‐MS/MS analysis. We chose a conservative approach of filtering each data set individually and concatenating the resulting filtered datasets, ensuring that only proteins represented in both datasets were used for downstream analyses and interpretations. Each sample group was initially filtered by excluding all mapped proteins that had any one of the following criteria: A minimum false discovery rate (*q*‐value) greater than 0.05, representation by fewer than two peptides, or three or fewer peptide spectral matches (PSMs). These criteria were selected based on proteomics field standard practices (Al‐Amrani et al. 2021).

### 4.6 Mapped Proteomics Analysis

To concatenate our results, a Venn analysis was performed to find the set of proteins identified as unambiguously present in both groups, resulting in a combined group (CG) data set. This data set reflected the shared mapped proteins between all eight (four control, four treatment) specimens in our sample. Groups 1 and 2 were further filtered to identify proteins with significant changes in protein expression between treatment and control specimens. First, proteins where at least one treatment specimen in each group had an abundance ratio adjusted *p* ≤ 0.05 were retained (*n* = 135). Then, proteins that had missing data for two or more specimen comparisons were discarded (*n* = 15). Those two data sets were subsequently concatenated via a Venn analysis to form a combined group significant (CGSig) data set. The result is that every protein in CGSig is significantly differentially expressed in at least one treatment/control comparison in Group 1 and at least one treatment/control comparison in Group 2.

Here, the abundance ratio is a normalized ratio of estimated protein abundance, based on peak intensity, for one proteomics sample over another proteomics sample, in our case a treatment specimen over a control specimen. Because protein abundances can vary significantly in terms of magnitude between samples in label free quantification, the estimated protein abundances are commonly transformed on a Log2 scale to normalize (adjust) them (Liu and Zhang 2021). To calculate the p‐value for adjusted abundance ratios *p* values we used a two‐tailed Students' *T*‐Test. One result of this transformation is that some calculated abundance ratios will effectively become ‐∞ or ∞ but are represented in the data set as values of −3.32 or 3.32. We calculated the average Log2 fold change reported in Table 1 by taking the mean of the estimated abundance ratios from samples that had significant *p* values, excluding significant samples that were equal to −3.32 or 3.32. For some samples, calculated means were close to 0 and/or removal of one outlier could change the sign (and thus interpretation) of the mean. In those cases, we do not provide an average value, instead we provided the number of specimens and their direction of fold change (up/down) (Table 1).

**Table 1:** List of proteins with significant differential expression between low dietary protein (10%) treatment and normal dietary protein (20%) control groups. Average Log2 fold change calculated by averaging of specimens with significant change of abundance ratio (SI 1). Up = significant treatment specimens have positive Log2 fold change meaning increased expression of that protein. Down = specimens have negative Log2 fold change meaning decreased expression of that protein. Proteins with blank averages are where a consistent signal was not identified due to individual variation. The number of specimens which could be individually interpreted as Up (U) or Down (D) is provided. Data are from Supporting Information.

Uniprot accession	Protein and gene name	GO: Process	Avg. Log2	Up/down
P63277	Amelogenin, X isoform GN=Amelx	Odontogenesis	−1.52	Down
O55188	Dentin matrix acidic phosphoprotein 1 GN=Dmp1	Odontogenesis		3U/2D
P97399	Dentin sialophosphoprotein GN=Dspp	Odontogenesis	1.77	Up
P43406	Integrin alpha‐V GN=Itgav	Osteogenesis	−2.66	Down
Q05117	Tartrate‐resistant acid phosphatase type 5 GN=Acp5	Osteogenesis	−1.71	Down
P28481	Collagen alpha‐1(II) chain GN=Col2a1	Osteogenesis		1U/2D
O54974	Galectin‐7 GN=Lgals7	Osteogenesis		3U/2D
P27005	Protein S100‐A8 GN=S100a8	Inflammation and immune response	−1.27	Down
O88947	Coagulation factor X GN=F10	Inflammation and immune response	−0.23	Down
Q9EPB4	Apoptosis‐associated speck‐like protein containing a CARD GN=Pycard	Inflammation and immune response		1U/1D
Q9JM83	Calmodulin‐4 GN=Calm4	Inflammation and immune response		2U/1D
Q9Z126	Platelet factor 4 GN=Pf4	Inflammation and immune response	0.92	Up
P16110	Galectin‐3 GN=Lgals3	Inflammation and immune response	1.10	Up
P07743	BPI fold‐containing family A member 2 GN=Bpifa2	Inflammation and immune response	1.50	Up
Q07456	Protein AMBP GN=Ambp	Inflammation and immune response	2.30	Up
Q61176	Arginase‐1 GN=Arg1	Inflammation and immune response	2.40	Up
Q9JJZ2	Tubulin alpha‐8 chain GN=Tuba8	Cytoskeleton formation	−2.29	Down
Q8R1Q8	Cytoplasmic dynein 1 light intermediate chain 1 GN=Dync1li1	Cytoskeleton formation	−1.59	Down
Q9CRB9	MICOS complex subunit Mic19 GN=Chchd3	Cytoskeleton formation	−1.17	Down
Q91ZU6	Dystonin GN=Dst	Cytoskeleton formation	−0.56	Down
P08032	Spectrin alpha chain, erythrocytic 1 GN=Spta1	Cytoskeleton formation		1U/1D
Q61554	Fibrillin‐1 GN=Fbn1	Cytoskeleton formation	0.09	Up
P15508	Spectrin beta chain, erythrocytic GN=Sptb	Cytoskeleton formation		1U/1D
P55292	Desmocollin‐2 GN=Dsc2	Cytoskeleton formation		2U/1D
P48193	Protein 4.1 GN=Epb41	Cytoskeleton formation		2U/1D
P10637	Microtubule‐associated protein tau GN=Mapt	Cytoskeleton formation	1.07	Up
O35902	Desmoglein‐3 GN=Dsg3	Cytoskeleton formation	1.20	Up
Q7TSF1	Desmoglein‐1‐beta GN=Dsg1b	Cytoskeleton formation	1.22	Up
P97350	Plakophilin‐1 GN=Pkp1	Cytoskeleton formation	1.58	Up
Q02257	Junction plakoglobin GN=Jup	Cytoskeleton formation	1.65	Up
E9Q557	Desmoplakin GN=Dsp	Cytoskeleton formation	1.80	Up
G5E8K5	Ankyrin‐3 GN=Ank3	Cytoskeleton formation	1.87	Up
Q3UV17	Keratin, type II cytoskeletal 2 oral GN=Krt76	Development of cornified envelope		3U/2D
A1L317	Keratin, type I cytoskeletal 24 GN=Krt24	Development of cornified envelope	0.76	Up
P04104	Keratin, type II cytoskeletal 1 GN=Krt1	Development of cornified envelope	1.26	Up
Q61781	Keratin, type I cytoskeletal 14 GN=Krt14	Development of cornified envelope	1.46	Up
P97347	Repetin GN=Rptn	Development of cornified envelope	1.47	Up
Q61414	Keratin, type I cytoskeletal 15 GN=Krt15	Development of cornified envelope	1.51	Up
Q09PK2	Retroviral‐like aspartic protease 1 GN=Asprv1	Development of cornified envelope	1.70	Up
Q62266	Cornifin‐A GN=Sprr1a	Development of cornified envelope	1.75	Up
A6BLY7	Keratin, type I cytoskeletal 28 GN=Krt28	Development of cornified envelope	1.89	Up
Q62267	Cornifin‐B GN=Sprr1b	Development of cornified envelope	2.93	Up
A2AQP0	Myosin‐7B GN=Myh7b	Muscle contraction	−2.37	Down
Q9QZ47	Troponin T, fast skeletal muscle GN=Tnnt3	Muscle contraction	−2.34	Down
Q5SX39	Myosin‐4 GN=Myh4	Muscle contraction	−2.25	Down
P13542	Myosin‐8 GN=Myh8	Muscle contraction	−2.24	Down
Q5SX40	Myosin‐1 GN=Myh1	Muscle contraction	−1.91	Down
P05977	Myosin light chain 1/3, skeletal muscle isoform GN=Myl1	Muscle contraction	−1.88	Down
P68134	Actin, alpha skeletal muscle GN=Acta1	Muscle contraction	−1.86	Down
Q3SX28	TREMBL:Q3SX28;Q5KR48 (*Bos taurus*) Tropomyosin 2	Muscle contraction	−1.61	Down
P13412	Troponin I, fast skeletal muscle GN=Tnni2	Muscle contraction	−1.59	Down
P97457	Myosin regulatory light chain 2, skeletal muscle isoformGN=Mylpf	Muscle contraction	−1.42	Down
P97447	Four and a half LIM domains protein 1 GN=Fhl1	Muscle contraction	−1.06	Down
A2ASS6	TitinGN=Ttn	Muscle contraction	−0.80	Down
Q9JI91	Alpha‐actinin‐2 GN=Actn2	Muscle contraction	−0.48	Down
P07310	Creatine kinase M‐type GN=Ckm	Phosphorylation	−2.03	Down
P28650	Adenylosuccinate synthetase isozyme 1 GN=Adssl1	Phosphorylation	−1.24	Down
Q9R0Y5	Adenylate kinase isoenzyme 1 GN=Ak1	Phosphorylation	−0.71	Down
P29699	Alpha‐2‐HS‐glycoprotein GN=Ahsg	Phosphorylation		2U/1D
Q9R0H0	Peroxisomal acyl‐coenzyme A oxidase 1 GN=Acox1	Regulation	−2.12	Down
P63330	Serine/threonine‐protein phosphatase 2A GN=Ppp2ca	Regulation	−1.78	Down
Q9EST5	Acidic leucine‐rich nuclear phosphoprotein 32 B GN=Anp32b	Regulation	−1.18	Down
P07759	Serine protease inhibitor A3K GN=Serpina3k	Regulation	−1.17	Down
P28665	Murinoglobulin‐1 GN=Mug1	Regulation	−0.88	Down
Q9Z2N8	Actin‐like protein 6A GN=Actl6a	Regulation	−0.62	Down
O55042	Alpha‐synuclein GN=Snca	Regulation	0.29	Up
Q5FWK3	Rho GTPase‐activating protein 1 GN=Arhgap1	Regulation		1U/1D
Q9D5V5	Cullin‐5 GN=Cul5	Regulation	0.38	Down
Q6P253	Dermokine GN=Dmkn	Regulation	0.89	Up
Q08189	Protein‐glutamine gamma‐glutamyltransferase E GN=Tgm3	Regulation	1.04	Up
Q60604	Adseverin GN=Scin	Regulation	1.32	Up
Q9D2Q8	Protein S100‐A14 GN=S100a14	Regulation	1.45	Up
Q8CGR5	Kallikrein‐14 GN=Klk14	Regulation	1.55	Up
P21570	Angiogenin GN=Ang	Regulation	2.42	Up
Q62203	Splicing factor 3A subunit 2 GN=Sf3a2	Transcription and repair	−0.21	Down
Q9R0Q6	Actin‐related protein 2/3 complex subunit 1A GN=Arpc1a	Transcription and repair	2.09	Up
P32848	Parvalbumin alpha GN=Pvalb	Binding	−2.24	Down
P62631	Elongation factor 1‐alpha 2 GN=Eef1a2	Binding	−2.01	Down
O35744	Chitinase‐like protein 3 GN=Chil3	Binding	−1.82	Down
P31786	Acyl‐CoA‐binding protein GN=Dbi	Binding	−1.72	Down
P81117	Nucleobindin‐2 GN=Nucb2	Binding	−1.09	Down
O70456	14‐3‐3 protein sigma GN=Sfn	Binding	1.09	Up
P63024	Vesicle‐associated membrane protein 3 GN=Vamp3	Transport	−3.07	Down
Q9JLM8	Serine/threonine‐protein kinase DCLK1 GN=Dclk1	Transport	−2.40	Down
P18872	Guanine nucleotide‐binding protein G(o) subunit alpha GN=Gnao1	Transport	−2.35	Down
Q8VEH3	ADP‐ribosylation factor‐like protein 8A GN=Arl8a	Transport	−2.18	Down
P61028	GN=Rab8b	Transport	−2.07	Down
P04247	Myoglobin GN=Mb	Transport	−1.71	Down
P02089	Hemoglobin subunit beta‐2 GN=Hbb‐b2	Transport	−1.51	Down
P04117	Fatty acid‐binding protein, adipocyte GN=Fabp4	Transport	−1.16	Down
P02088	Hemoglobin subunit beta‐1 GN=Hbb‐b1	Transport		1U/1D
P04919	Band 3 anion transport protein GN=Slc4a1	Transport		1U/1D
P97449	Aminopeptidase N GN=Anpep	Transport		1U/1D
P14602	Heat shock protein beta‐1 GN=Hspb1	Transport	1.44	Up
P24668	Cation‐dependent mannose‐6‐phosphate receptor GN=M6pr	Transport	2.37	Up
Q8VHI3	GDP‐fucose protein O‐fucosyltransferase 2 GN=Pofut2	Metabolism	−2.35	Down
Q5SWU9	Acetyl‐CoA carboxylase 1 GN=Acaca	Metabolism	−2.03	Down
Q9D2R0	Acetoacetyl‐CoA synthetase GN=Aacs	Metabolism	−1.93	Down
O70503	Very‐long‐chain 3‐oxoacyl‐CoA reductase GN=Hsd17b12	Metabolism	−1.87	Down
P19096	Fatty acid synthase GN=Fasn	Metabolism	−1.83	Down
P21550	Beta‐enolase GN=Eno3	Metabolism	−1.77	Down
O70250	Phosphoglycerate mutase 2 GN=Pgam2	Metabolism	−1.75	Down
Q8R429	Sarcoplasmic/endoplasmic reticulum calcium ATPase 1 GN=Atp2a1	Metabolism	−1.73	Down
Q9EQ06	Estradiol 17‐beta‐dehydrogenase 11 GN=Hsd17b11	Metabolism	−1.38	Down
P13634	Carbonic anhydrase 1 GN=Ca1	Metabolism	−0.92	Down
Q9WUB3	Glycogen phosphorylase, muscle form GN=Pygm	Metabolism	−0.87	Down
Q9CQT1	Methylthioribose‐1‐phosphate isomerase GN=Mri1	Metabolism	−0.25	Down
P00920	Carbonic anhydrase 2 GN=Ca2	Metabolism		1U/1D
O89020	Afamin GN=Afm	Metabolism		1U/1D
O09131	Glutathione S‐transferase omega‐1 GN=Gsto1	Metabolism	1.22	Up
P97370	Sodium/potassium‐transporting ATPase subunit beta‐3 GN=Atp1b3	Metabolism	1.22	Up
P47739	Aldehyde dehydrogenase, dimeric ZDP‐preferring GN=Aldh3a1	Metabolism	1.48	Up
Q9QYJ0	DnaJ homolog subfamily A member 2 GN=Dnaja2	Metabolism	2.00	Up
Q9R099	Transducin beta‐like protein 2 GN=Tbl2	Unknown	−1.52	Down
Q00898	Alpha‐1‐antitrypsin 1‐5 GN=Serpina1e	Unknown	−1.25	Down
Q9D7B7	Probable glutathione peroxidase 8 GN=Gpx8	Unknown		1U/2D
P00687	Alpha‐amylase 1 GN=Amy1	Unknown		3U/2D
Q8CIT9	Suprabasin GN=Sbsn	Unknown	1.28	Up
Q9Z331	Keratin, type II cytoskeletal 6B GN=Krt6b	Unknown	1.30	Up
Q8CF02	Protein FAM25C GN=Fam25c	Unknown	1.81	Up

To identify protein functions, associated interactions, and general biological profiles represented in CG and CGSig we performed Gene Ontology (GO) enrichment analysis via the ClusterProfiler tool of ProteoRE and Pathway Enrichment Analysis via REACTOME (Croft et al. 2011; Wu et al. 2021). For GO enrichment analyses, we queried at two ontology levels, using cutoffs for *p* value of 0.05 and *q* value of 0.05. Outputs for GO analyses were used to assign broad categorical function to proteins (Table 1) based on Metabolic Functions, Cellular Component, and Biological Processes categories (Figure 3).

For pathway enrichment analysis via REACTOME we queried the *Mus musculus* REACTOME for the proteins found in the CGSig data set. To calculate enrichment, the number of entities (in our case proteins) identified as belonging within a specific pathway are identified. Then the total number of entities (proteins) that could be contained in that pathway is calculated and divided by the total number of entities (proteins) from the organism (in our case *Mus*). The resulting “Ratio” is used to correct for pathway size to determine which pathways are overrepresented compared to a random distribution. A pathway is considered “enriched” when the input from the data set (x) is a higher proportion of the total number of entities (*n*) than would be expected by chance. The resulting probability estimate is represented by a *p* value calculated on a 95% confidence interval and pathways with Entities *p* ≤ 0.05 are significantly enriched. Enriched pathways were then ranked based on the Entity Ratio, which represents the percentage of all *Mus* proteins in that pathway with a significant enrichment in our analysis (i.e., an Entity Ratio of 0.04 represents 4% of all *Mus* proteins known in that pathway). Pathways with higher entity ratios are ranked higher within Table 2.

**Table 2:** Significantly enriched REACTOME pathways derived from CGSig data set (120 proteins). Entities Ratio is the number of proteins from pathway name/total number of proteins for *Mus musculus* in REACTOME database. Entities pValue is hypothesis test of chances of the number of proteins found being within pathway compared to random, significant *p* value = enriched pathway. Accession numbers of proteins from CGSig which belong to a pathway, not an inclusive list of all proteins known within the pathway.

**REACTOME Pathway identifier**	**Pathway name**	**Proteins found in pathway**	**Total number of proteins in pathway**	**#Reactions found**	**#Reactions total**	**Entities ratio**	**Entities *p* value**	**Uniprot accession of significantly differentially expressed proteins (CGSig) belonging in pathway**
R‐MMU‐1266738	Developmental Biology	19	573	22	218	0.0520	0.0000	P97350; P04104; Q8CGR5; Q61414; Q3UV17; P15508; P97347; P28481; Q9R0Q6; Q61781; P43406; P08032; Q02257; Q9Z331; O35902; P55292; E9Q557; Q9JJZ2; A6BLY7
R‐MMU‐6798695	Neutrophil degranulation	14	532	8	10	0.0483	0.0020	P29699; P97350; P04104; P97449; Q61176; P43406; P16110; Q8VEH3; Q02257; Q9JM83; Q9EPB4; E9Q557; Q8R1Q8; P27005
R‐MMU‐6805567	Keratinization	13	191	14	26	0.0173	0.0000	P97350; P04104; Q8CGR5; Q61414; Q3UV17; P97347; Q61781; Q02257; Q9Z331; O35902; P55292; E9Q557; A6BLY7
R‐MMU‐397014	Muscle contraction	9	180	12	43	0.0163	0.0002	Q9JI91; Q8R429; Q9QZ47; P05977; P68134; P97457; P13412; P97370; P13542
R‐MMU‐5357801	Programmed cell death	5	151	6	155	0.0137	0.0253	P97350; O70456; P10637; O35902; E9Q557
R‐MMU‐381426	Regulation of insulin‐like growth factor (IGF) transport and uptake by Insulin‐like growth factor binding proteins (IGFBPs)	4	123	1	6	0.0112	0.0462	P29699; Q61554; O55188; P63277
R‐MMU‐8957275	Posttranslational protein phosphorylation	4	117	1	1	0.0106	0.0397	P29699; Q61554; O55188; P63277
R‐MMU‐109581	Apoptosis	5	110	6	110	0.0100	0.0073	P97350; O70456; P10637; O35902; E9Q557
R‐MMU‐1169410	Antiviral mechanism by IFN‐stimulated genes	4	101	3	44	0.0092	0.0251	P63330; P10637; O55042; Q9JJZ2
R‐MMU‐6807878	COPI‐mediated anterograde transport	4	100	6	12	0.0091	0.0243	P08032; P15508; Q9JJZ2; Q8R1Q8
R‐MMU‐1500931	Cell‐Cell communication	4	100	5	56	0.0091	0.0243	Q91ZU6; P21570; Q02257; Q61781
R‐MMU‐6809371	Formation of the cornified envelope	13	98	7	19	0.0089	0.0000	P97350; P04104; Q8CGR5; Q61414; Q3UV17; P97347; Q61781; Q02257; Q9Z331; O35902; P55292; E9Q557; A6BLY7
R‐MMU‐373760	L1CAM interactions	4	76	3	24	0.0069	0.0099	P43406; P08032; P15508; Q9JJZ2
R‐MMU‐446728	Cell junction organization	4	76	5	40	0.0069	0.0099	Q91ZU6; P21570; Q02257; Q61781
R‐MMU‐9013106	RHOC GTPase cycle	3	75	2	6	0.0068	0.0493	P63024; Q02257; Q5FWK3
R‐MMU‐9013026	RHOB GTPase cycle	3	74	2	6	0.0067	0.0477	P63024; Q02257; Q5FWK3
R‐MMU‐9833482	PKR‐mediated signaling	4	71	3	28	0.0064	0.0078	P63330; P10637; O55042; Q9JJZ2
R‐MMU‐3371497	HSP90 chaperone cycle for steroid hormone receptors (SHR) in the presence of ligand	3	71	4	22	0.0064	0.0431	Q9QYJ0; Q9JJZ2; Q8R1Q8
R‐MMU‐75105	Fatty acyl‐CoA biosynthesis	3	70	4	20	0.0064	0.0417	P19096; Q5SWU9; O70503
R‐MMU‐9013406	RHOQ GTPase cycle	3	62	2	5	0.0056	0.0308	P63024; Q02257; Q5FWK3
R‐MMU‐3000178	ECM proteoglycans	4	52	7	14	0.0047	0.0026	O55188; P43406; P97399; P28481
R‐MMU‐75153	Apoptotic execution phase	4	50	4	53	0.0045	0.0023	P97350; P10637; O35902; E9Q557
R‐MMU‐375165	NCAM signaling for neurite out‐growth	3	45	4	8	0.0041	0.0134	P08032; P15508; P28481
R‐MMU‐390522	Striated muscle contraction	6	39	4	4	0.0035	0.0000	Q9JI91; Q9QZ47; P05977; P68134; P13412; P13542
R‐MMU‐111465	Apoptotic cleavage of cellular proteins	4	38	4	38	0.0034	0.0008	P97350; P10637; O35902; E9Q557
R‐MMU‐1237044	Erythrocytes take up carbon dioxide and release oxygen	3	24	3	8	0.0022	0.0024	P00920; P13634; P04919
R‐MMU‐1480926	O2/CO2 exchange in erythrocytes	3	24	5	14	0.0022	0.0024	P00920; P13634; P04919
R‐MMU‐140875	Common pathway of fibrin clot formation	2	24	6	29	0.0022	0.0286	O88947; Q9Z126
R‐MMU‐1475029	Reversible hydration of carbon dioxide	2	17	2	8	0.0015	0.0151	P00920; P13634
R‐MMU‐1247673	Erythrocytes take up oxygen and release carbon dioxide	3	16	2	6	0.0015	0.0008	P00920; P13634; P04919
R‐MMU‐445095	Interaction between L1 and ankyrins	2	12	1	1	0.0011	0.0078	P08032; P15508
R‐MMU‐351906	Apoptotic cleavage of cell adhesion proteins	3	11	3	10	0.0010	0.0003	P97350; O35902; E9Q557
R‐MMU‐446107	Type I hemidesmosome assembly	2	9	2	4	0.0008	0.0045	Q91ZU6; Q61781
R‐MMU‐5660668	CLEC7A/inflammasome pathway	1	4	2	3	0.0004	0.0426	Q9EPB4

### 4.7 Preliminary Test of Developmental Archive

Prior research and the design of our study allowed us to conduct a preliminary investigation of whether enamel and dentin proteomes represent an archive of protein expression during mineralization, rather than proteins expressed earlier in tooth development or at the time of specimen collection. We queried the Mouse Gene eXpression Database (GXD) to determine if a subset of proteins was expressed at developmental stages before the onset of mineralization, during mineralization, or after mineralization was complete. First, we investigated proteins known to be expressed during tooth mineralization (Pandya et al. 2017). The study of Pandya et al. (2017) reported 24 proteins present during tooth mineralization, we identified 20 of those 24 proteins in our CG data set. We also investigated proteins related to odontogenesis/osteogenesis, immune, and actin‐based myosins from our CGSig data set. A protein's associated gene being expressed during mineralization, but not earlier or later in time, would support the argument that tooth proteomes represent a specific window of development during amelogenesis. The table containing the proteins queried in the GXD is within Supporting Information.

## 5 Results

### 5.1 Phenotyping

**Figure 2:**
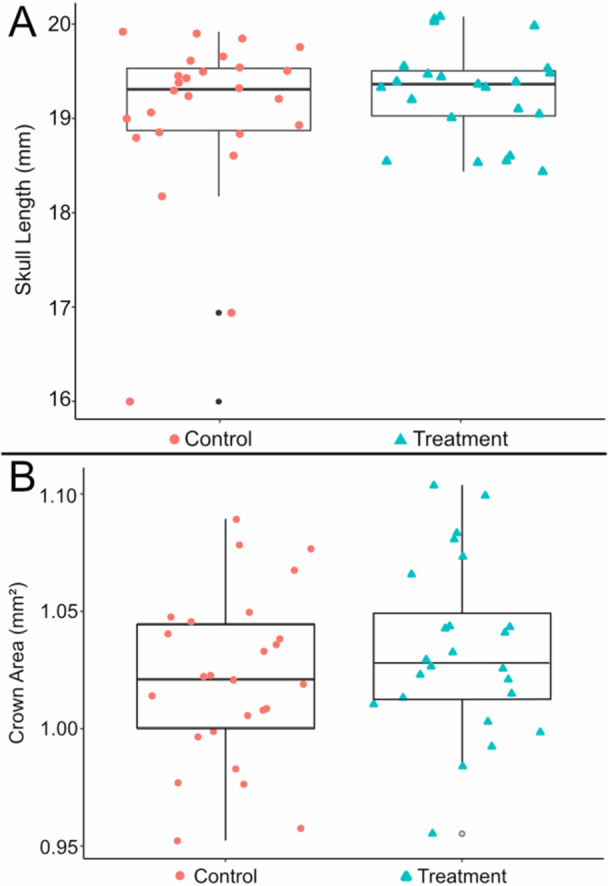
Results of phenotypic investigation. (A) Estimates of straight line skull length. (B) Estimates of crown area.

We found no significant differences in size between treatment and control groups (Table 3). Mean skull length for the treatment group was 19.20 mm and control group was 19.03 mm (Figure 2A). Mean crown area for treatment group was 1.03 mm^2^ and control group mean was 1.02 mm^2^ (Figure 2B).

**Table 3:** Welch's two sample *T*‐tests to test for significant difference in mean between treatment and control group crown area and skull length.

Crown area by group			
	*t* = −1.1575	*df* = 47.474	*p* = 0.8736
95% confidence intervals	−0.02914971	Infinity	
Mean control	1.021672 mm^2^		
Mean treatment	1.033573 mm^2^		
Skull length by group			
	*t* = −86423	*df* = 41.17	*p* = 0.8038
95% confidence intervals	−0.5085681	Infinity	
Mean control	19.02755 mm		
Mean treatment	19.20011 mm		

### 5.2 Proteomics

Within G1, a total of 2189 unique proteins were mapped (Supplemental Materials). One thousand six hundred and twenty‐two unique proteins were mapped for G2 (Supporting Information). The combined group (CG) of proteins shared between G1 and G2 was 1469 unique proteins (Supporting Information). Of the combined group, there were a total of 120 proteins with significant differential expression (fold change) (CGSig; Table 1; Figures 4 and S1–S6).

**Figure 3:**
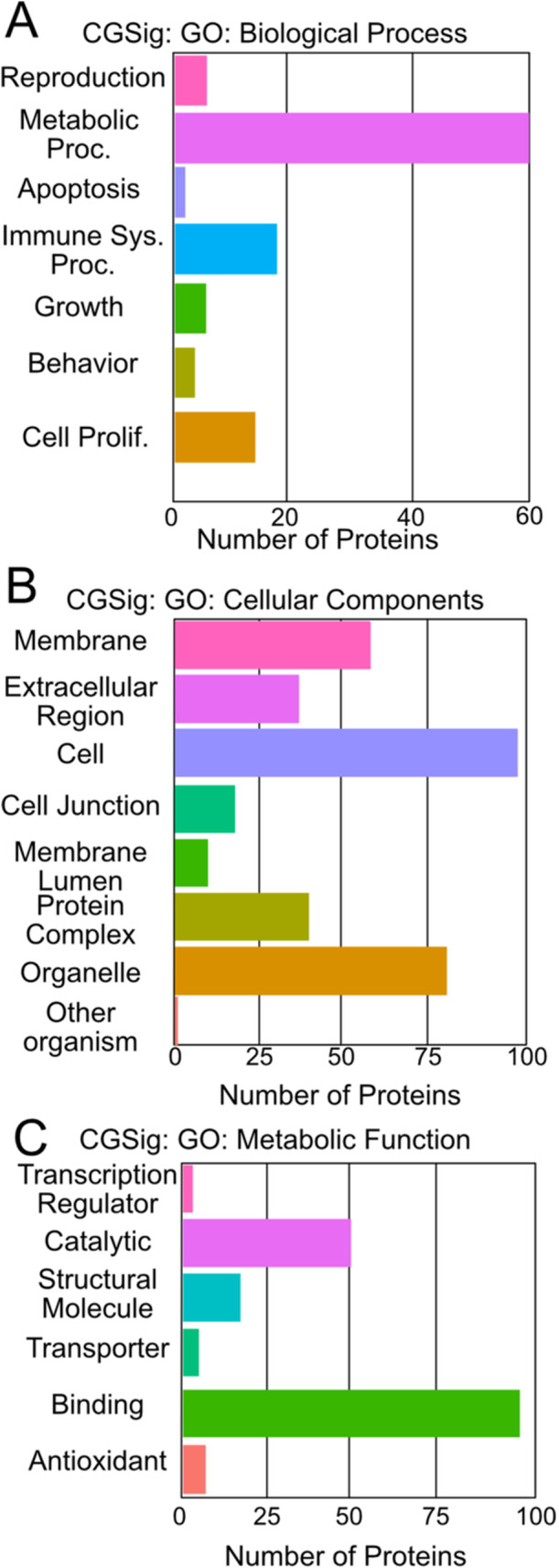
Most enriched pathways from gene ontology (GO) analysis of CGSig. (A) Biological processes, (B) cellular components, (C) metabolic function.

Gene ontology profiling revealed that both CG and CGSig are comprised of sets of proteins primarily associated with the Binding (in Molecular Function), the Cell generally (as opposed to a specific cellular component), and the Biological Process of Metabolism (Figure 3; Supporting Information). Pathway enrichment analysis via REACTOME identified 387 biological pathways associated with significantly differentially expressed proteins (Supplemental Materials). Of those 387, 34 were significantly enriched (*p *≤ 0.05) (Table 2). Approximately 47% of matched proteins are represented in the top six enriched pathways (Entity Ratio ≥ 0.01) (Table 2; Supporting Information).

### 5.3 Odontogenesis and Osteogenesis Proteins

**Figure 4:**
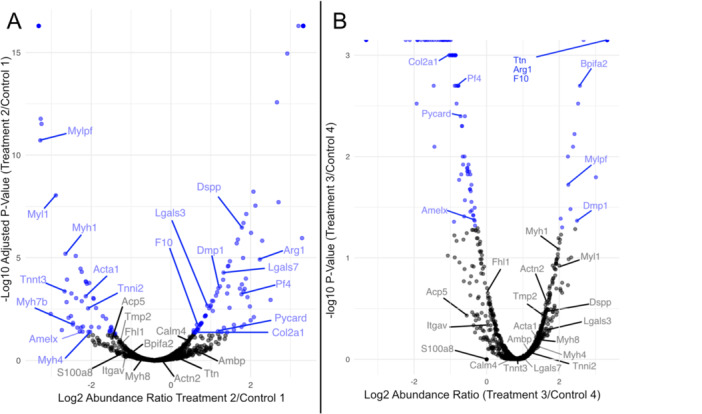
Volcano of specimen proteomics data exemplified by selected comparison from each group. (A) From Group 1 Treatment 2 over Control 1. (B) From Group 2 Treatment 3 over Control 4. Blue dots show Adjusted Abundance Ratio *p* ≤ 0.05. Labeled dots are proteins related to osteogenesis, odontogenesis, inflammation and immune response, and muscle contraction. Volcano plots of remaining 6 comparisons are provided in Supporting Information.

A total of eight proteins in CG were identified as belonging to biomineralization. Seven of them had significant differential expression (Log2 fold change) between treatment and control groups (Table 1; Figure 4). In low‐protein treatments, the major enamel‐forming protein, Amelogenin X (AMELX) had an average −1.52‐fold change in expression. One of the two major dentin‐forming proteins, Dentin sialophosphoprotein (DSPP) had a 1.77‐fold increase in expression. The other dentin‐forming protein, Dentin matrix acidic phosphoprotein 1 (DMP1) did not have a clear direction of differential expression; three treatment comparisons showed an increase in protein expression, while two had a decrease in expression. For osteogenesis, Integrin alpha‐V (ITGAV) had −2.66‐fold change and Tartrate‐resistant acid phosphatase type 5 (ACP5) had −1.71‐fold change. The remaining two osteogenic proteins Collagen alpha‐1(II) chain (COL2A1) and Galectin‐7 (LGALS7) had variation that made interpretation unclear. For COL2A1, one specimen had increased and two had decreased expression. For LGALS7, three were increased and two decreased.

### 5.4 Inflammation and Immune Response

A total of 229 proteins were associated with Immune System Response. Nine proteins associated with inflammation and immune system response had significant differential expression (Log2 fold change) between treatment and control groups (Table 1; Figure 4). Two of the nine proteins, S100‐A8 (S100A8) and Coagulation factor X (F10), had a −1.27 and −0.23 fold change in treatment groups, respectively. Five of the nine proteins had increases in expression: 0.92 for Platelet factor 4 (PF4), 1.10 for Galectin‐3 (LGALS3), 1.50 for BPI fold‐containing family A member 2 (BPIFA2), 2.30 for Protein AMBP (AMBP), and 2.40 for Arginase‐1 (ARG1). Two of the immune proteins, Apoptosis‐associated speck‐like protein containing a CARD (PYCARD) and Calmodulin‐4 (CALM4), lacked consistent signal between pairwise specimen comparisons. For PYCARD, one specimen showed increased expression and one showed decreased expression. For CALM4, two specimens were increased in expression and one decreased in expression.

### 5.5 Muscle Contraction

A total of 42 proteins were associated with muscle contraction and 13 of those proteins had significant differential expression. All of them had decreased expression for treatment specimens (Table 1; Figure 4). Seven of the 13 proteins are actin‐based myosins (Myosins 1, 4, 7B, 8, Myosin Light chain 1, Tropomyosin 2, and Myosin regulatory light chain 2), with the remaining six being actin‐specific proteins (Troponin T, Actins Alpha 1 and Alpha 2, Titin, and Four and a half LIM domains protein 1).

### 5.6 Preliminary Test of Developmental Archive

Genes associated with two of 20 tooth proteins previously found during mineralization (Pandya et al. 2017) are also found in tooth tissues before the onset of amelogenesis based on a query of GXD. Those genes are *Alpl* and *Itgb1*. Both are found only in the tooth developmental stage immediately before amelogenesis (TS21 associated with embryonic days 12.5–14). The genes associated with the remaining 18 proteins are found only in developmental stages associated with molar amelogenesis (TS22+, embryonic day 15 through postnatal day 8). Querying genes associated with our proteomic sample's differentially expressed immune and actin‐based myosin proteins within GXD revealed that immune genes are universally expressed within tooth tissues during all reported stages until adulthood. Actin‐based myosins were present from the onset of mineralization forward. Two of the actin‐based myosins (*Actn2* and *Fhl1*) were reported as definitively absent from tooth tissues at E14.5 (i.e., just before mineralization) (Visel 2004). Additionally, one gene (*Myh1*) was present in mineralization stages but was definitively absent from postnatal week 6–8 aged mouse specimens (Freeman et al. 1998).

## 6 Discussion

### 6.1 Phenotyping

Our hypotheses were that specimens in the treatment group would have reduced skull length and M1 size, results we did not recover. Both phenotypic results were unexpected because many studies have reported variation in skull size associated with nutritional quality in rats, mice, and pigs (e.g., Barbeito‐Andrés et al. 2016; Dickerson and Hughes 1972; Lobe et al. 2006; McCance et al. 1961; Miller and German 1999; Pucciarelli 1980, 1981), and four previous studies reported variation in the size of the M1 due to dietary changes (Paynter and Grainger 1956; Holloway et al. 1961; Paynter 1961; Shaw and Griffiths 1963). Why then did we not recover similar signals?

First, we note that these previous studies sampled specimens at different ontogenetic stages than we did, most sampled specimens that were older than ours (40‐days postnatal to 10‐month postnatal), a single study (Barbeito‐Andrés et al. 2016) sampled specimens younger than ours (embryonic day 18 to postnatal day 1). Considering skull length, mice grow continuously until they reach sexual maturity and rapidly between birth and weaning. It is possible that any differences in skull length were simply not manifested at the ontogenetic stage we sampled. This does not explain, however, the lack of difference in M1 size reported previously, because M1s are determinate in growth and cease to grow by postnatal day 8 (Ungar 2010; Pandya et al. 2017). In comparison to our study design using 10% dietary protein as the low‐protein treatment, some other studies used dietary protein quantity as low as 8% (e.g., Paynter and Grainger 1956; Holloway et al. 1961; Paynter 1961; Shaw and Griffiths 1963; Miller and German 1999). This small additional reduction in dietary protein may be sufficient to trigger effects. Finally, we recognize that different dental and cranial traits are impacted by dietary protein more readily than others. Cranial traits have been reported to be less impacted by reduced dietary protein than postcranial traits (e.g., McCance et al. 1961; Lobe et al. 2006) and M1 size may be less impacted than other traits of the M1 (e.g., angle of cusps) (Paynter and Grainger 1956; Holloway et al. 1961; Paynter 1961).

At present we can only conclude that there is no difference in M1 size or skull length of specimens from our groups. However, our proteomic results (discussed in detail below) inform us that development of the tooth was perturbed in ways that likely impact other aspects of tooth phenotype. For instance, changes in relative enamel or dentin thickness would not be recovered by our estimates of size, because these are internal structural changes to tooth composition. Future more comprehensive phenotyping efforts may reveal significant differences.

### 6.2 Developmental Archive

Results from GXD queries support the argument that our tooth proteome data set primarily represents gene expression during mineralization and not before mineralization. For example, genes for actin‐based myosins of interest within our data set are not expressed in mouse teeth before the onset of amelogenesis. While it is not possible to say whether the measured immune‐system proteins in our proteomic sample were expressed during mineralization or at the time of euthanasia, previous studies (e.g., Green et al. 2019; Jágr et al. 2019) have identified immune and inflammation related proteins incorporated in tooth tissues, either within enamel itself or within other tissues of the enamel‐dentin junction, such as salivary glands. In our case, we recover several of the immune related proteins reported by those studies, including S100A8 and CALM4, potentially supporting the idea that molar enamel represents an archive of gene and protein expression during amelogenesis. This conclusion is further supported by the fact that the gene for differentially expressed MYH1 in our data set is not expressed after mineralization, indicating our MYH1 signal likely represents protein expression during mineralization rather than a later time point (Freeman et al. 1998). Further, we would not expect expression of amelogenesis‐specific genes or proteins after the end of mineralization, because of the cessation of proliferation of ameloblasts and lack of vascularization within the fully mineralized tooth (Alghadeer et al. 2023; Nanci 2007). While this hypothesis requires further directed experimental validation, our results support the concept that the recovered tooth proteome represents a limited window of development.

### 6.3 Odontogenic Proteins

We predicted that proteins associated with enamel and dentin formation would be altered by our feeding experiment, and specifically that they would be decreased in expression. The major enamel forming protein, AMELX, was significantly reduced in expression for treatment specimens (Table 1; Figure 4). We anticipated that our dietary protein reduction would result in decreased tooth size, associated with decreased AMELX expression, because AMELX is a necessary component for the formation of enamel. While changes in AMELX met our expectations, measures dentin forming protein expression and lower M1 size did not.

In the case of DMP1, there is not a clear signal to interpret whether expression was increased or decreased in our proteomics sample. This highlights the challenges of drawing interpretations from proteomic data where individual variation can influence the overall interpretation. This challenge is recognized by the field of quantitative proteomics, but still represents an area where increased research efforts will be needed (Al‐Amrani et al. 2021; Chantada‐Vazquez et al. 2022; Liu and Zhang 2021; Steward et al. 2023). A clearer interpretation of DMP1 expression, would be helpful for constructing future hypotheses. For instance, decreased expression of DMP1 should lead to decreased expression of DSPP and dentin hypomineralization, suggesting that DMP1 and DSPP expression contribute significantly to dentinogenesis imperfecta (Orsini et al. 2014; Shi et al. 2020). Being able to robustly identify such patterns or, at minimum, make supported interpretations based on the variable evidence, will enhance the utility of future quantitative proteomic studies.

Our finding of increased DSPP expression seemed initially counterintuitive. However, this result is supported by a recent study of protein expression in a hypomineralized enamel defect found in humans (Mukhtar et al. 2022). In this study of hypomineralized molars, the enamel defect impacts the first permanent molars of children and results in a significant reduction in mineral density from normal teeth (Mukhtar et al. 2022). This reduced density was associated with downregulation of AMELX, upregulation of DSPP, but no reported differences in DMP1 expression (Mukhtar et al. 2022).

The causal mechanism for the pattern of increased DSPP expression is unknown. The general role of DSPP is to control the conversion of dental pulp cells into odontoblasts via binding with Integrin beta 6 (ITGB6) (Ritchie 2018; Wan et al. 2016). Previous work indicated that mice with either a DSPP heterozygous (DSPP^+/^^−^) or DSPP knockout (DSPP^−^^/^^−^) genotypes experience dentin dysplasia and dentinogenesis imperfecta due to haploinsufficiency of DSPP (Shi et al. 2020). Haploinsufficiency suggests that dentin is impacted when DSPP expression is decreased but does not indicate what phenotype results from elevated DSPP expression. Amelogenesis imperfecta enamel is typically thin and chalky while dentin appears to be normally mineralized, suggesting that DSPP overexpression does not result in dentin hypermineralization, but this has not been experimentally validated and dentin structure was not reported by Mukhtar et al. (2022).

Our proteomic results appear consistent with protein expression patterns associated with amelogenesis imperfecta and not dentinogenesis imperfecta, based on the shared expression changes for AMELX and DSPP (Mukhtar et al. 2022; Orsini et al. 2014; Shi et al. 2020). It is unlikely that both amelogenesis and dentinogenesis imperfecta are simultaneously present within a single specimen's dentition. Only a single study has reported compounded presence of amelogenesis and dentinogenesis imperfecta, which occurred in an MSX2 knockout transgenic line (Aïoub et al. 2007).

It is possible that the signal here is due to experimental design of this study, where enamel and dentin are pooled together. The concern is that dentin constitutes a larger amount of the overall tooth and could “swamp” the proteomic signal. This concern might be further compounded by the relatively small enamel proteome (e.g., Jágr et al. 2019; Gil‐Bona and Bidlack 2020; Castiblanco et al. 2015). However, this “swamping” of proteome signals from nondentin tissue appears unlikely in our sample due to consistent signals of expression changes for many proteins not related to dentin. In cases where a set of proteins “swamp” the signal, it is due to exceptionally high abundance of certain proteins. For example, raw blood is dominated by ~20 proteins related to hemoglobin, swamping out other protein signals (Molloy et al. 2023). In our case, recovery of 1469 proteins and 120 significantly different proteins associated with numerous processes suggests that our signal is not driven by one tissue or category of proteins within the pooled tissue sample.

It remains possible that the overall strength of expression for enamel‐related proteins is under‐estimated because of our sampling approach. In general, a stronger estimate could come from optimizations of protocols intended for recovery of specific proteins, that is, a switch to Data Dependent Acquisition (DDA) from our Data Independent Acquisition (DIA) approach. In general, when DDA methods are compared to DIA there is recovery of the same or additional differences between groups, an increase in the measured strength of differences in expression between groups, and increased confidence in those differences, but there is also a decrease in the number of distinct proteins recovered (Gent et al. 2024; Guo and Huan 2020; Molloy et al. 2023). In other words, DDA increases signal of specific proteins at the trade‐off of decreased coverage of a given proteome, a result that DIA provides (Gent et al. 2024; Guo and Huan 2020). We predict that future micro‐sampling of enamel, i.e., optimization of extraction for only the enamel proteome, would produce a stronger signal of the same enamel protein expression differences we already recovered with our DIA approach. However, this hypothesis remains to be tested.

Thus, we conclude that our measures of differentially expressed amelogenesis‐associated proteins indicate that the development of thinner and less mineralized dental enamel could be a phenotypically plastic response to reduced dietary protein during early development. Such a result would help explain why there are disparate signals between proteomic and phenotypic results: while tooth size is not affected, tooth or microstruture composition may be.

This result would also be in line with previously reported phenomenon of Linear Enamel Hypoplasia (LEH). Arrest or reduction of enamel formation due to changes in diet are hypothesized to form the characteristic banding of LEH (Skinner 2023). Many studies have attributed formation of LEH in humans to less access to high quality diets during tooth development and even proposed that such changes may be heritable in the context of epigenetics (Amoroso et al. 2014; Lawrence et al. 2021). If such traits are heritable, investigation of modifications of dental specific proteins (such as histone modifications or posttranslational modifications) may be fruitful avenues of future research. Thus, a combination of phenotyping and proteomics could reveal the mechanistic basis for this phenomenon. At present we cannot say whether our samples have enamel disruptions that are characteristic of LEH without further investigation. However, we would expect teeth from samples fed a low protein diet to have both poorly mineralized dental enamel and evidence of LEH. To test these hypotheses, it is necessary to utilize synchrotron scanning or destructive sampling in the form of histology. However, because of the structure of our study we can assess if our specimens possess characteristics of LEH and/or amelogenesis imperfecta, while simultaneously having controlled for their early diets during tooth development.

### 6.4 Immune and Inflammation Proteins

Previous work on mapping protein expression across micro‐sampled enamel sections of pig molars had suggested that there was possibility of recovering immune and inflammation related proteins from mineralized dentition (Green et al. 2019). In their case, Green et al. (2019) were constructing a detailed map of proteomic expression associated with amelogenesis. Their reported immune system proteins were localized from enamel which came from along the enamel‐dentin junction (Green et al. 2019). Their study was not designed nor attempted to induce differential protein expression based on experimental procedures. In our case, we were unsure if we would recover immune and inflammation proteins because we created a tissue‐averaged signal by crushing and processing the entire M1. By finding these proteins in our proteomics sample and recovering differential expression of them, we present a novel result of immune response to an environmental change. Of the nine immune or inflammation response proteins with significant fold change, five were increased in expression for treatments relative to controls, two were reduced in expression, and two had mixed interpretations (Table 1).

Seven of the nine proteins are primarily associated with neutrophil degranulation, including transport and proliferation of neutrophils. Of these seven, five were increased in expression. However, Calmodulin 4 (CALM4) had a mixed interpretation and coagulation factor X (F10) was reduced in expression. Neutrophils function as critical, but specialized, immune system response cells. Neutrophils contain granules of multiple types (azurophilic, specific, ficolin‐rich, tertiary, and secretory), that target specific threats and/or regulate immune system response to specific infectious threats (Eichelberger and Goldman 2020; Othman et al. 2022; Yin and Heit 2018).

The precise nature of what was being targeted by immune system activation is unknown. However, the result of F10 being reduced in expression may provide some insight into the potential infectious threat. Deficiency of F10 has been implicated as part of an immune response to the common, antibiotic‐resistant, bacterium *Acinetobacter baumannii* (Choby et al. 2019). In those cases, F10 deficiency is indicative of an increased abundance of neutrophils and macrophages (Choby et al. 2019). Thus, though F10 is decreased in expression, relative to the increase in five other neutrophil degranulation proteins, the combination of patterns supports a conclusion that our treatment group had higher immune system response than our control group. Future research efforts to systematically compare immune‐related proteomic signatures from mineralized structures to standardized health monitoring tools could prove fruitful.

We also recovered changes in PYCARD and S100A8 within our proteomics sample, that are indicative of an inflammation response (Table 1). Studies have indicated that PYCARD directly mitigates inflammation when upregulated and contributes to inflammation when downregulated (Sartoretto et al. 2019; Wittmann et al. 2021). In our sample, one treatment specimen showed a moderate increase in PYCARD expression over controls, and one showed a moderate decrease (Table 1). Importantly, deviation in either direction suggests that there is either an increased response to inflammation (increased expression) or increased inflammation present (decreased expression) (Sartoretto et al. 2019; Wittmann et al. 2021). Reduced expression is recovered for S100A8, consistent with an ongoing physiological response to inflammation (S. Wang et al. 2018). These patterns of differential expression may represent increased inflammation response in some of the treatment specimens, as compared to control specimens. Due to the limited nature of our data and potential variation in inflammation response between specimens, these results should be interpreted cautiously.

Because of our controlled experimental design, we postulate that reduced dietary protein is the cause of increased inflammation. A recent study investigating the impact of low dietary protein during gestation indicates that intrauterine inflammation can occur and result in increased inflammation present in the offspring of Syrian golden hamsters (Mohammed et al. 2023). While Mohammed et al. (2023) focused on measuring inflammation of the liver of the offspring, the connection between low dietary protein during embryonic and postnatal development and increased inflammation was strongly established. Our study and results further support this connection. Future studies should aim to systematically investigate proteomic signals of mineralized structures along with standardized inflammation panels. This would further establish the connection between dietary protein, inflammation, and the signals archived within mineralized structures.

### 6.5 Muscle Contraction Proteins

All thirteen significantly modified Muscle Contraction pathway proteins were reduced in expression. They were predominantly actin‐based myosins, which play a critical role in cellular movement and structure formation by acting as motor molecules (Guhathakurta et al. 2018). Actin‐based myosins are broadly implicated in the proper development of many tissues, including dentition (Du et al. 2024; Guhathakurta et al. 2018; Luis and Schnorrer 2021). During dental development actin‐based myosins contribute to the proper formation of enamel rods by transporting ameloblasts (Duverger and Morasso 2018). Lower expression of actin‐based myosins, including some of those that are differentially expressed in our study (e.g., Myosin‐1, Myosin‐4, Myosin‐8, and Tropinin 1) are associated with the syndromic form of amelogenesis imperfecta (Duverger and Morasso 2018). To date, no proteomic study of dentition has recovered differential expression of actin‐based myosins, but none of the previous dental proteomic studies attempted to experimentally induce differential expression of these proteins. Future investigations should center on understanding the distribution of actin‐based myosins within the tooth and the correlation between protein expression in teeth and other tissues when dietary‐protein is reduced.

### 6.6 Potential Pathways for Phenotypic Plasticity

We recovered 34 significantly enriched REACTOME pathways from our 120 significantly differentially expressed proteins. Among those pathways, two of the top six most enriched pathways, regulation of insulin‐like growth factor and muscle contraction, have proteins that play many roles during morphogenesis.

Insulin‐like growth factor plays a role in a number of different developmental processes, including odontogenesis, bone development, organ development, and brain development (Baroncelli et al. 2017; Brown et al. 2017; Chen et al. 2009; Dodington et al. 2021; Luo et al. 2020; Montivero et al. 2021; Oyanagi et al. 2019; Vassilakos et al. 2019). Thus, regulation of IGF is a complex set of processes with many different factors involved in regulating expression in different tissues. This includes regulation by thyroid growth hormone (Yakar and Isaksson 2016) and regulation via phosphorylation of binding proteins to aid in transport, activation, and inhibition (Chrudinova et al. 2024; Dong et al. 2022; Huttlin et al. 2010; Palma‐Lara et al. 2023; Tagliabracci et al. 2015). Given the importance of IGF to multiple developmental processes, regulation of IGF is a probable candidate for a mechanism underlying phenotypic plasticity. Experimental manipulation of IGF1 and IGF2 during odontogenesis has revealed systematic changes to the size and number of cusps of developing molars (Oyanagi et al. 2019). The connection between IGF1 and IGF2 gene expression and proteins associated with IGF regulation, which includes Amelogenin X, is not currently well understood (Bansal et al. 2012; Oyanagi et al. 2019; Pandya and Diekwisch 2021). However, regulation of insulin‐like growth factor offers a promising pathway for future investigation, particularly in elucidating responses of IGF gene expression and associated regulatory factors to environmental perturbations, such as poor diet.

Similarly, our collection of proteins from the muscle contraction pathway are primarily actin‐based myosins. The function of myosins as motor proteins is well documented, but the specific nature of how myosins interact during odontogenesis is poorly understood (Du et al. 2024; Guhathakurta et al. 2018). Previous proteomic studies of teeth have not reported differential expression of actin‐based myosins, making this result unexpected. Simultaneously, those studies were not attempting to experimentally induce plastic responses like our study. The connection of actin‐based myosins with body size and potentially with amelogenesis imperfecta suggests that future investigations may be fruitful. These future studies would hopefully confirm the results we recovered and further explore the relationship between protein expression and phenotypic plasticity.

## 7 Conclusions

Proteomic expression results supported our prediction that halving dietary protein during embryogenesis and early postnatal development would alter expression of proteins recovered from mouse molars. Specifically, we identified 120 differentially expressed proteins associated with a reduction of dietary protein during embryonic and early postnatal development. Changes in dietary protein have been proposed to result in phenotypically plastic changes in molar size and skull length. Our study did not recover significant differences between normal and low protein groups for either a measure of molar size or skull length. The discordance between positive proteomic results and negative phenotypic results suggests that phenotypic differences may be more nuanced that initially predicted.

We recovered significant changes in proteins associated with dental development, that are primarily within the pathway associated with regulation of Insulin‐Like Growth Factor. The connection between IGF and dental proteins remains to be further investigated, but changes in expression in this pathway could directly influence tooth structure. We also identified systematically reduced expression for proteins in the Muscle contraction pathway, specifically actin‐based myosins, a novel discovery for tooth‐derived proteomics data. Actin‐based myosins are broadly implicated in vertebrate development and are correlated with dental and general body development. Taken together, it is possible that we perturbed tooth phenotypes, such as enamel or dentin thickness or density, that are not captured by our measures of craniodental size.

Fortunately, the study presented here is part of a larger research program aimed at more comprehensive phenotyping efforts. The results of this study will allow us to focus on investigating phenotypic hypotheses derived from our proteomics results (e.g., testing for amelogenesis imperfecta). As well as continuing to investigate other aspects of craniodental morphology, to include assessment of size of the second and third molars and shape of all three molars, along with cranial traits such as depth and width of the cranium. Inclusion of these more comprehensive phenotypic data may recover differences between groups that were not recovered from the data reported here.

Our controlled feeding experiment also induced increased immune system activation and inflammation response, as evidenced by increased expression of proteins in the Neutrophil degranulation pathway. While this result does not directly inform us about phenotypic plasticity, that we could potentially to derive such information from a fully mineralized teeth could prove useful for studying the biology of deceased or extinct organisms. New studies aimed at microsampling enamel, dentin, and along the enamel‐dentin junction to determine both the source of these signals and which tissues contain them are necessary.

Finally, we propose that proteomic quantification of nonexperimental organisms will prove fruitful and predict that dietary changes in wild settings will change enamel and dentin formation by altering aspects of the IGF regulatory pathway and potentially expression of actin‐based myosins. Future research efforts then should focus on elucidating the connection between IGF gene expression and enamel and dentin protein expression; determining the role of actin‐based myosins in tooth and skeletal development; and comparing the immune and inflammation signals in proteomic data to classically used tools (e.g., blood panels and cortisol screening). With a combination of phenotyping and proteomic investigations it will become clearer precisely what traits are more likely to be perturbed, and what sections of these pathways are causal to those changes. In turn, this will enhance the utility of quantitative proteomics for investigating organismal biology more broadly.

## Ethics Statement

Live animal experiments were conducted in accordance with guidelines and regulations from Stony Brook University Institutional Animal Care and Use Committee (IACUC). Research was conducted in accordance with plan and procedures in approved protocol SBU IACUC 2023‐0014.

## Consent

The authors have nothing to report.

## Conflicts of Interest

The authors declare no conflicts of interest.

## Supporting information


**Supplemental Figure S1:** Treatment 1 Over Control 1. **Supplemental Figure S2:** Treatment 1 over Control 2. **Supplemental Figure S3:** Treatment 2 over Control 2. **Supplemental Figure S4:** Treatment 3 over Control 3. **Supplemental Figure S5:** Treatment 4 over Control 3. **Supplemental Figure S6:** Treatment 4 over Control 4.

## Data Availability

All mapped and analyzed proteomics data and phenotypic data analyzed, along with R‐scripts and ProteoRE workflow are included as Supplemental Materials. These Supplemental Materials are available via Dryad Digital Repository DOI: 10.5061/dryad.kh18932j0. Raw proteomics data are available on the MassIVE database with Dataset Identifier: MSV000098600.
