# Supplementary material for: Reduced Dietary Protein Induces Changes in the Dental Proteome
Source: J Exp Zool B Mol Dev Evol. 2026 Jan 8;346(2):107–27. doi: 10.1002/jezb.70004 (PMC12887919; doi:10.1002/jezb.70004)

Supplemental Figures:

Volcano Plots from Burroughs et al. (In Review) Tooth Proteomics

Low-dietary protein (10%) sample specimen over control (20% protein) sample specimen. Blue dots show adjusted p-value is ≤ 0.05. Labeled proteins refer to Table 1 in Burroughs et al. (In Review).

Supplemental Figure S1: Treatment 1 Over Control 1


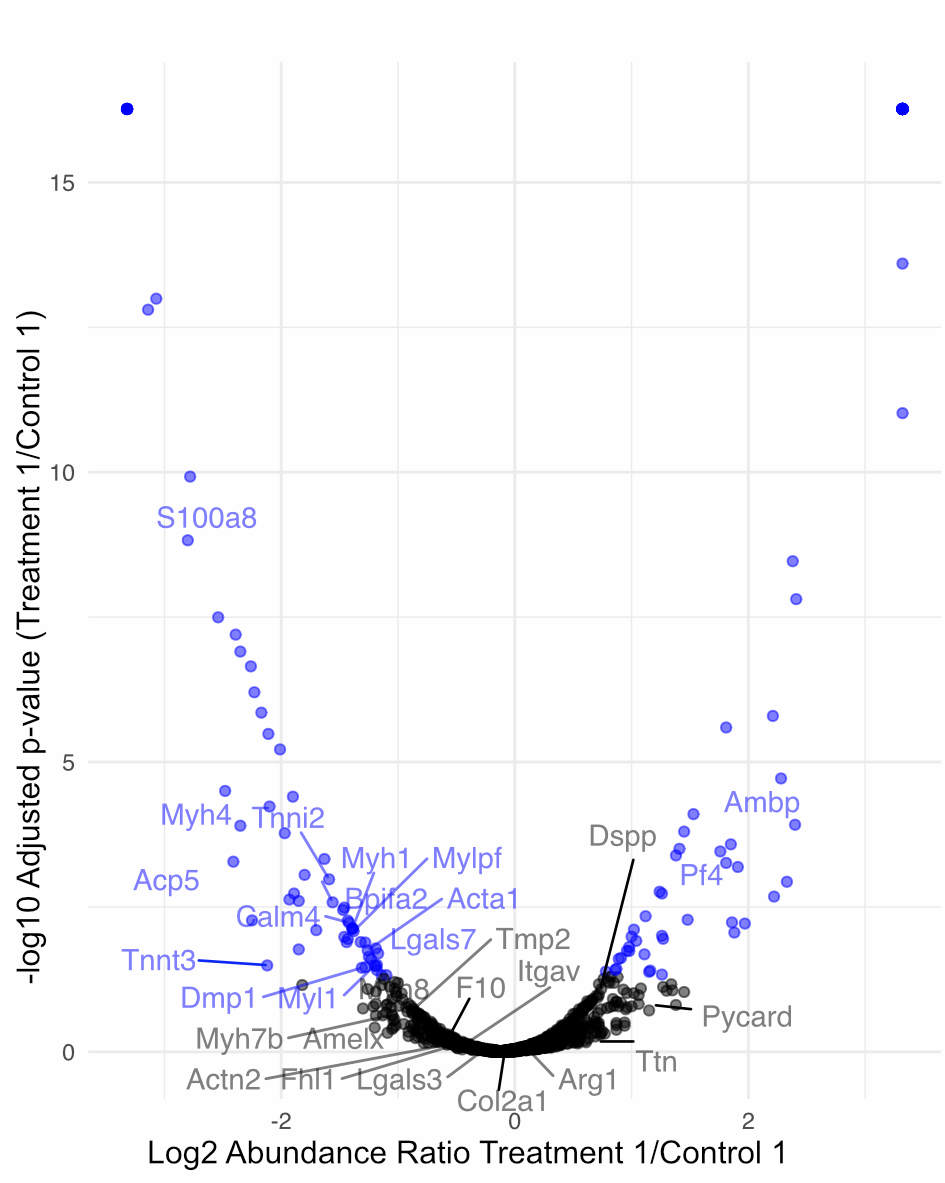


Supplemental Figure S2: Treatment 1 over Control 2


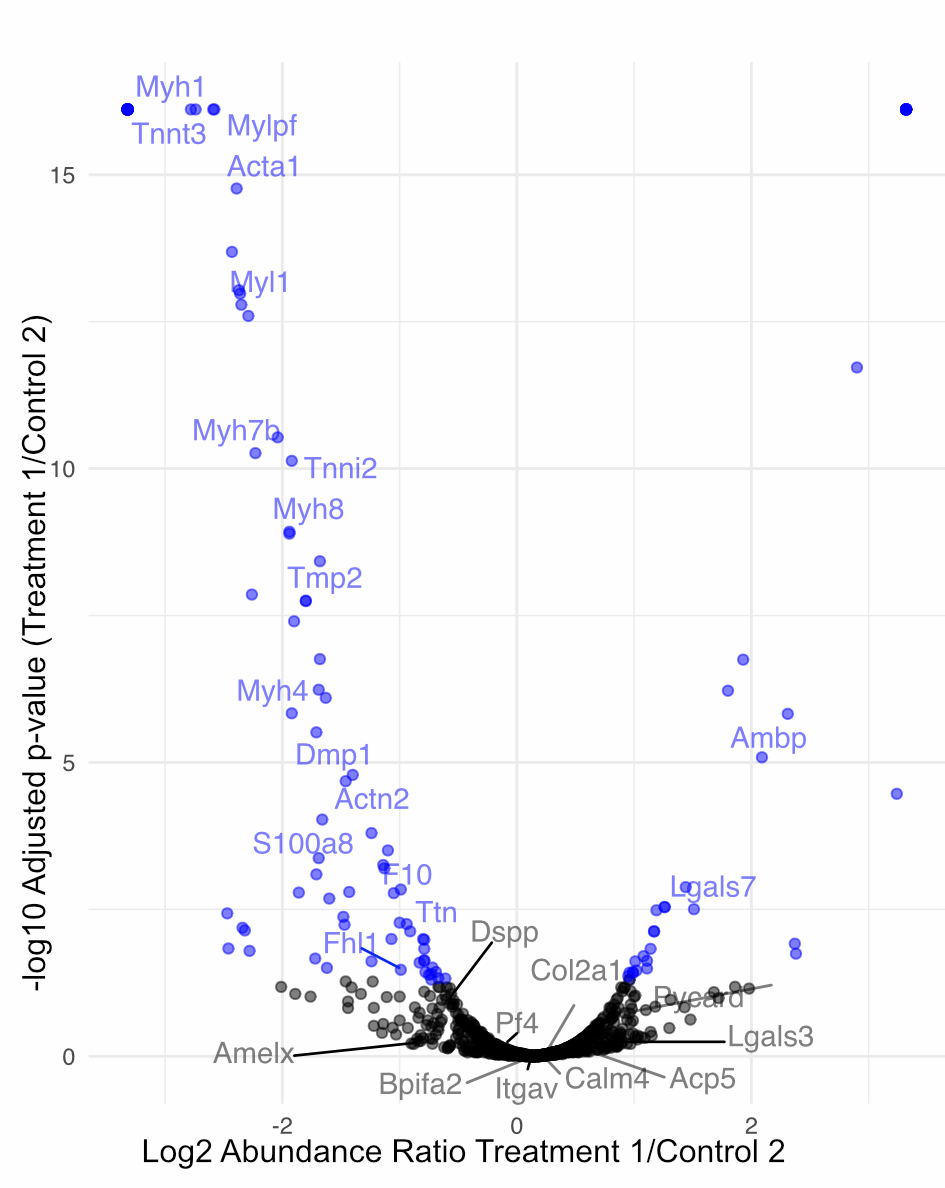


Supplemental Figure S3: Treatment 2 over Control 2


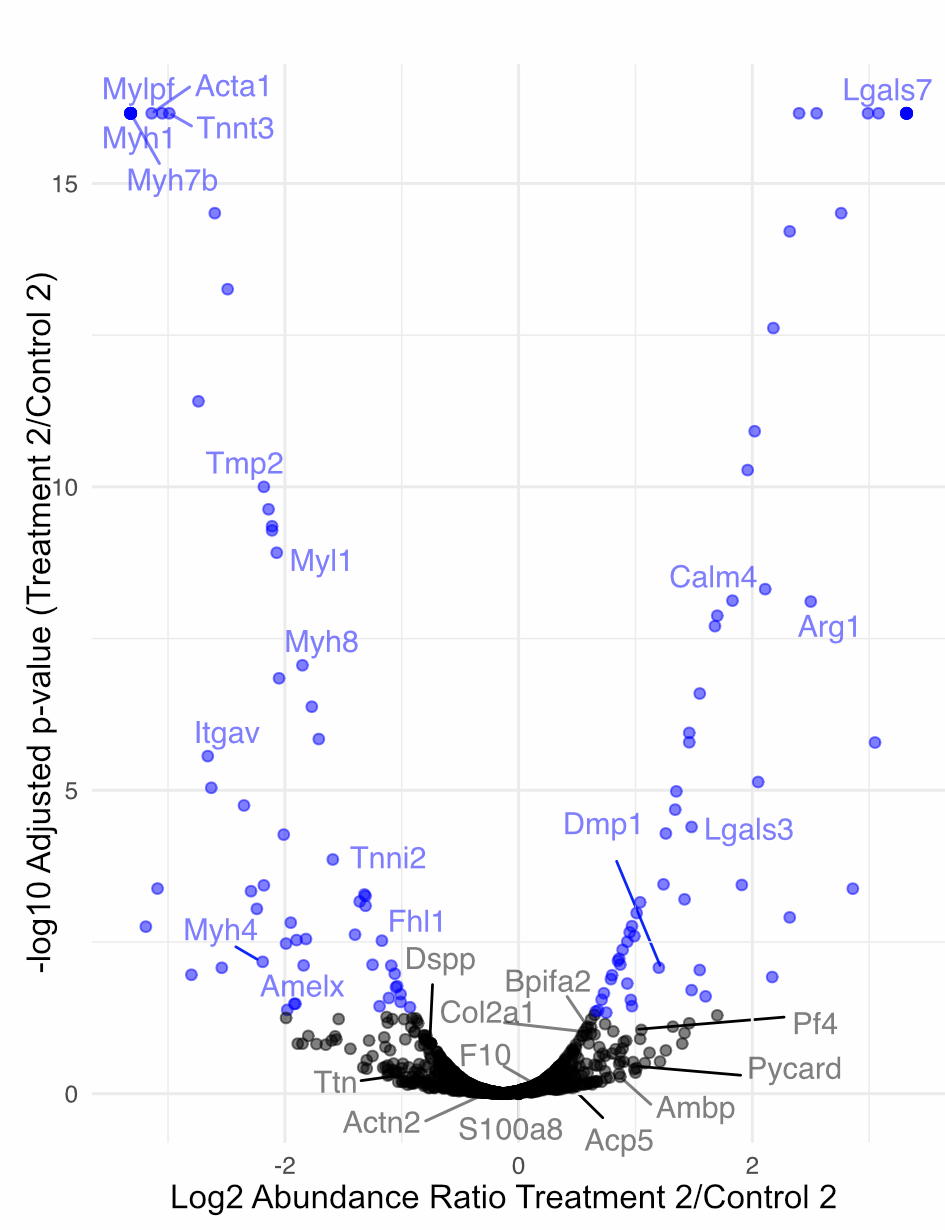


Supplemental Figure S4: Treatment 3 over Control 3


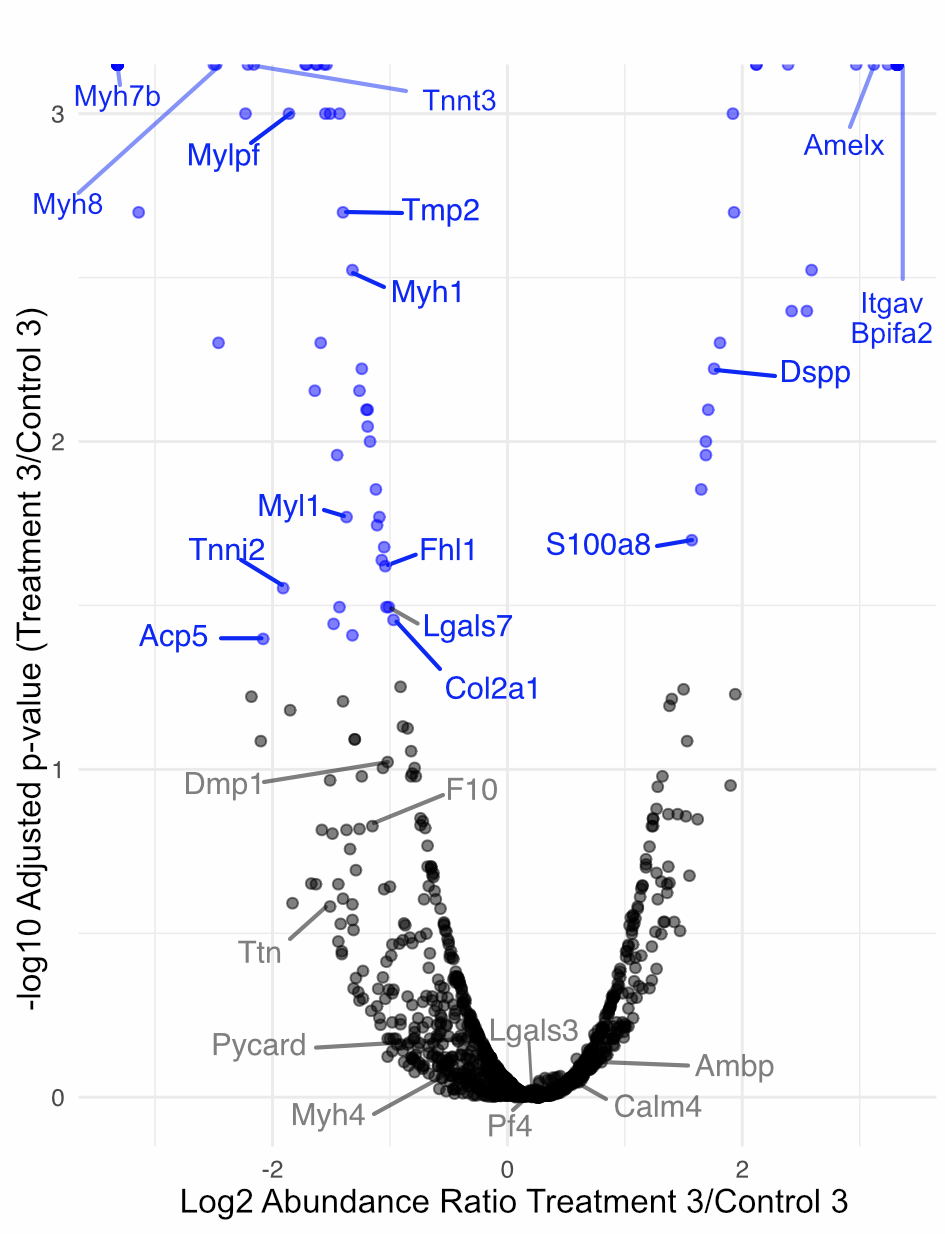


Supplemental Figure S5: Treatment 4 over Control 3


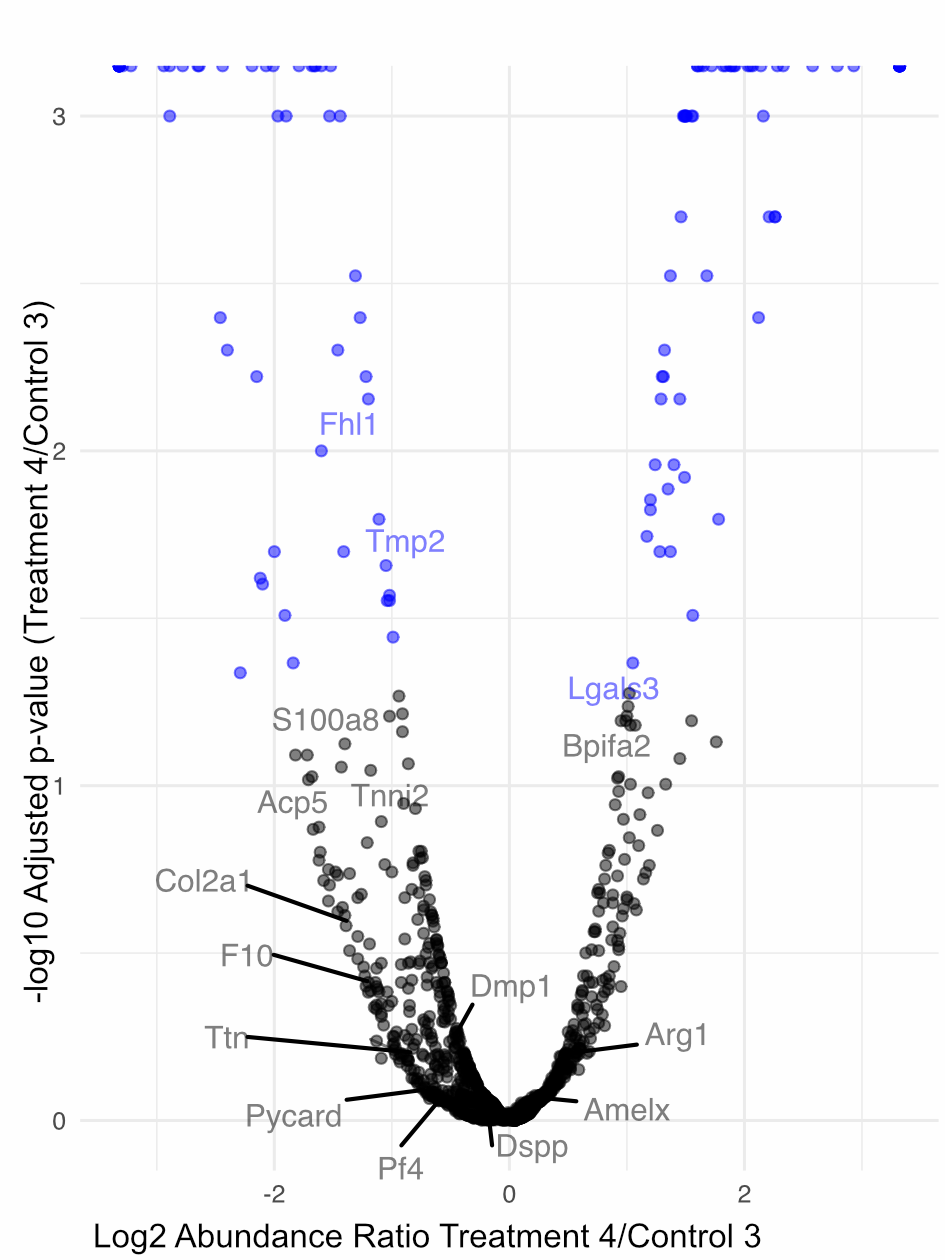


Supplemental Figure S6: Treatment 4 over Control 4


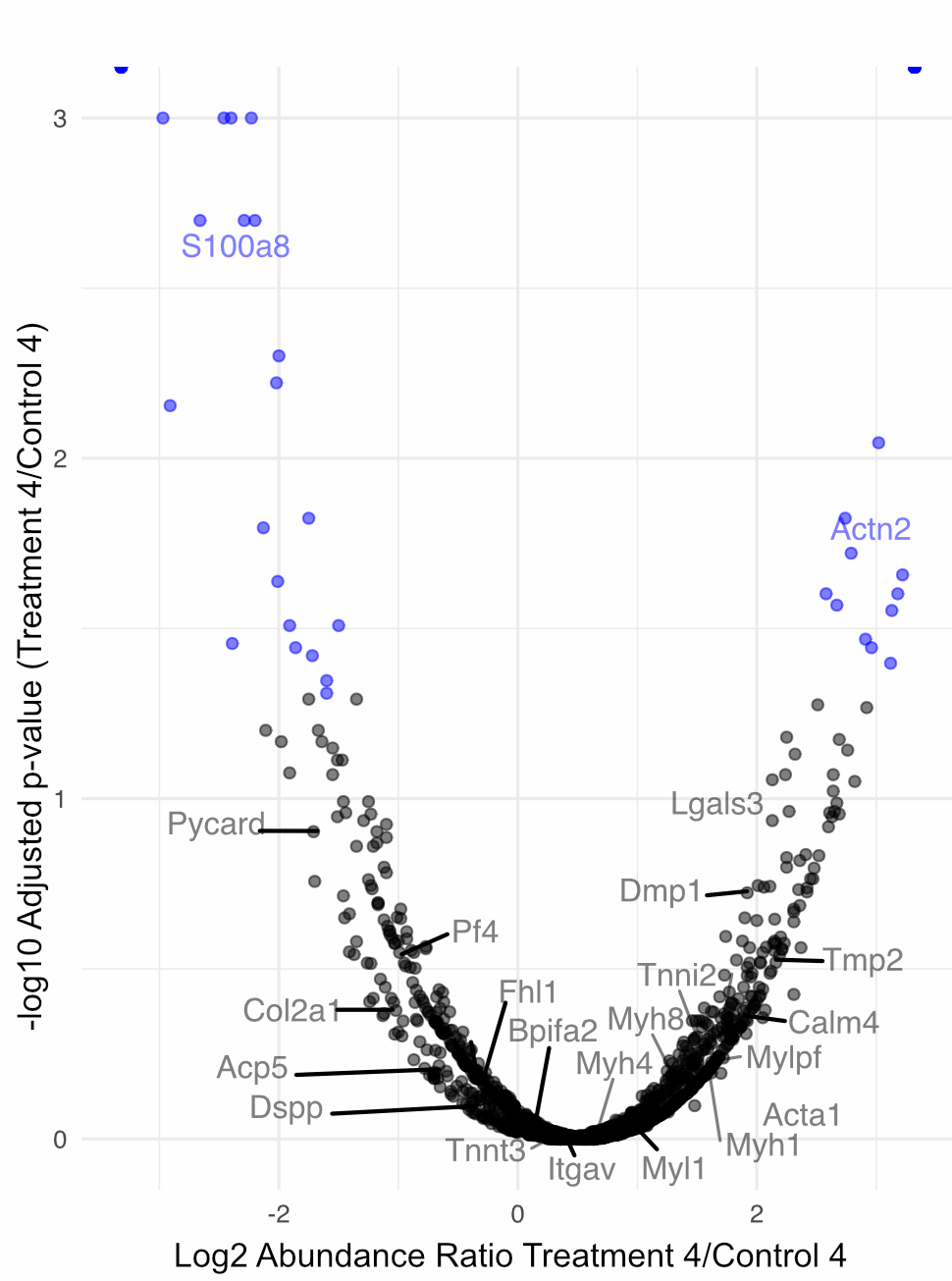

Supplement: Supplementary file 1 — Supplemental Figure S1: Treatment 1 Over Control 1. Supplemental Figure S2: Treatment 1 over Control 2. Supplemental Figure S3: Treatment 2 over Control 2. Supplemental Figure S4: Treatment 3 over Control 3. Supplemental Figure S5: Treatment 4 over Control 3. Supplemental Figure S6: Treatment 4 over Control 4. [file JEZ-346-107-s001.docx]
